# A *Drosophila* model of dominant inclusion body myopathy type 3 shows diminished myosin kinetics that reduce muscle power and yield myofibrillar defects

**DOI:** 10.1242/dmm.028050

**Published:** 2017-06-01

**Authors:** Jennifer A. Suggs, Girish C. Melkani, Bernadette M. Glasheen, Mia M. Detor, Anju Melkani, Nathan P. Marsan, Douglas M. Swank, Sanford I. Bernstein

**Affiliations:** 1 Department of Biology and Molecular Biology Institute, San Diego State University, San Diego, CA 92182-4614, USA; 2 Department of Biological Sciences and Center for Biotechnology and Interdisciplinary Studies, Rensselaer Polytechnic Institute, Troy, NY 12180-3590, USA

**Keywords:** Inclusion body myopathy type 3, Myosin heavy chain, *Drosophila melanogaster*, Myofibril, Muscle mechanics

## Abstract

Individuals with inclusion body myopathy type 3 (IBM3) display congenital joint contractures with early-onset muscle weakness that becomes more severe in adulthood. The disease arises from an autosomal dominant point mutation causing an E706K substitution in myosin heavy chain type IIa. We have previously expressed the corresponding myosin mutation (E701K) in homozygous *Drosophila* indirect flight muscles and recapitulated the myofibrillar degeneration and inclusion bodies observed in the human disease. We have also found that purified E701K myosin has dramatically reduced actin-sliding velocity and ATPase levels. Since IBM3 is a dominant condition, we now examine the disease state in heterozygote *Drosophila* in order to gain a mechanistic understanding of E701K pathogenicity. Myosin ATPase activities in heterozygotes suggest that approximately equimolar levels of myosin accumulate from each allele. *In vitro* actin sliding velocity rates for myosin isolated from the heterozygotes were lower than the control, but higher than for the pure mutant isoform. Although sarcomeric ultrastructure was nearly wild type in young adults, mechanical analysis of skinned indirect flight muscle fibers revealed a 59% decrease in maximum oscillatory power generation and an approximately 20% reduction in the frequency at which maximum power was produced. Rate constant analyses suggest a decrease in the rate of myosin attachment to actin, with myosin spending decreased time in the strongly bound state. These mechanical alterations result in a one-third decrease in wing beat frequency and marginal flight ability. With aging, muscle ultrastructure and function progressively declined. Aged myofibrils showed Z-line streaming, consistent with the human heterozygote phenotype. Based upon the mechanical studies, we hypothesize that the mutation decreases the probability of the power stroke occurring and/or alters the degree of movement of the myosin lever arm, resulting in decreased *in vitro* motility, reduced muscle power output and focal myofibrillar disorganization similar to that seen in individuals with IBM3.

## Abstract (precis)

**Summary:** Reduced muscle power output and progressive myofibrillar defects in a *Drosophila* model of inclusion body myopathy 3 arise from the decreased rate of weak to strong actin-binding transition of myosin.

## INTRODUCTION

Inclusion body myopathy type 3 (IBM3) is a rare autosomal dominant disease caused by a mutation in the *MYH2* gene that results in an E706K substitution in fast muscle myosin heavy chain IIa (Darin et al., 1998; Martinsson et al., 2000). In general, joint contractures are observed at birth, resolving to mild myopathy during childhood and progressing to muscle weakness during middle age. Muscle biopsies reveal progressive histopathological abnormalities, including focal myofilament disruption, Z-line streaming and rimmed vacuoles with 15-20 nm diameter inclusion bodies (Tajsharghi et al., 2002).

The charge change in IBM3 myosin is within the evolutionarily conserved SH1-SH2 alpha helix of the motor domain (Martinsson et al., 2000; Wang et al., 2012). This short, kinked and highly flexible alpha helix contains the SH1 (Cys-707) and SH2 (Cys-697) cysteines, which are ∼1.9 nm apart, at opposite ends of the helix (Bobkova et al., 1999; Rayment et al., 1993). The SH1-SH2 helix plays a key role in myosin conformational changes during force generation (Huston et al., 1988; Preller et al., 2011). Crosslinking the cysteine residues reduces myosin ATPase rates and weakens actin affinity of the myosin molecules (Thompson et al., 2008). Mutations in the SH1-SH2 helix region can depress the mechanochemical activity of the myosin motor (Hu et al., 2002; Kad et al., 2007; Preller et al., 2011; Zeng et al., 2004). Crystallographic analysis indicates that the SH1-SH2 helix melts following the power stroke to yield an internally uncoupled state (Himmel et al., 2002). Our previous molecular modeling suggested that this state might be stabilized by the E706K mutation, resulting in a reduced ability to proceed through the mechanochemical cycle (Wang et al., 2012).

Investigation into the mechanism of E706K myosin pathogenicity has been hampered by a paucity of biopsied muscle and is further complicated by the presence of other myosin isoforms in mutant skeletal muscle fibers. To clarify the means by which the IBM3 mutation causes muscle dysfunction, we developed an IBM3 disease model in *Drosophila melanogaster* (Wang et al., 2012). *Drosophila* is an advantageous system for the study of myosin myopathies (Swank et al., 2000), because a single gene coupled with alternative RNA splicing yields all muscle myosin heavy chain isoforms (Bernstein et al., 1983; Rozek and Davidson, 1983). Introduction of myosin transgenes and subsequent genetic manipulation permits expression of mutant myosin heavy chains in the absence of wild-type isoforms in either all or in subgroups of fly muscles.

Exclusive expression of the corresponding E701K IBM3 myosin mutation in *Drosophila* fast-twitch indirect flight muscles (IFMs) enabled us to define defects associated with the genetic lesion. Homozygous expression recapitulated hallmarks of the disease: muscle fibers contained myofibrils that progressively degenerated and rimmed vacuoles containing inclusions (Wang et al., 2012). Biochemical analysis of purified E701K myosin revealed an 80% reduction in actin-activated ATPase activity and *in vitro* actin filament sliding velocity (Wang et al., 2012). Furthermore, motor domains of isolated, full-length mutant myosin molecules were thermally unstable, suggesting that aggregated myosin may be implicated in inclusion body formation (Wang et al., 2012).

Here, we take advantage of the *Drosophila* system to explore the genetically dominant basis of IBM3 myosin pathogenicity in humans, and to gain insights into the disease mechanism through biochemical, ultrastructural and fiber mechanical studies. Our ATPase assays on myosin isolated from *E701K*/*+* IFMs suggest the accumulation of equimolar levels of wild-type and mutant myosins. Despite relatively normal ultrastructure, flight ability is dramatically impaired in young *E701K*/*+* organisms. Mechanical analysis of skinned heterozygous IFM fibers reveals severely decreased muscle power generation. Evaluation of our data indicates that slowed *E701K*/*+* muscle fiber kinetics result from a reduced overall rate of cross-bridge cycling that arises from slower steps associated with actin binding and the power stroke. We interpret our results in light of the observed effects on active muscle stiffness and indicate how these changes may be implicated in the morphological and functional deterioration of muscles in individuals with IBM3.

## RESULTS

Heterozygotes were generated by crossing *E701K-3* or *E701K-5* homozygous flies that exclusively express IBM3 (E701K) mutant myosin heavy chain in their IFMs and jump muscles to *yw* ‘wild-type’ organisms. The transgenes are expressed in the *Mhc^10^* genetic background, which lacks muscle myosin in these muscle types (Collier et al., 1990). Homozygotes were crossed to *yw* flies, such that the progeny had one endogenous wild-type *Mhc* allele and one transgene expressing E701K myosin heavy chain. Similarly, we crossed females from a control line (Swank et al., 2000) expressing a transgenic source of wild-type myosin in the *Mhc^10^* background (*w*, *PwMhc2*; *Mhc^10^*) with *yw* male flies to generate control heterozygotes (*w*, *PwMhc2*/*y*; *Mhc^10^*/*+*, hereafter referred to as *PwMhc2*/+). The heterozygous mutant fly lines lack a statistically significant relative difference in levels of myosin expression [104±5.7% (±s.e.m.) for *E701K-*3/+; 92.9±9.0% for *E701K-*5/+] compared with age-matched *PwMhc2*/*+* control heterozygotes (100±5.8%) and served as the basis of the current study (Student's *t*-test, *P*>0.5).

### ATPase activity assays indicate that E701K and wild-type myosin accumulate at equimolar levels in heterozygotes

**Fig. 1.**
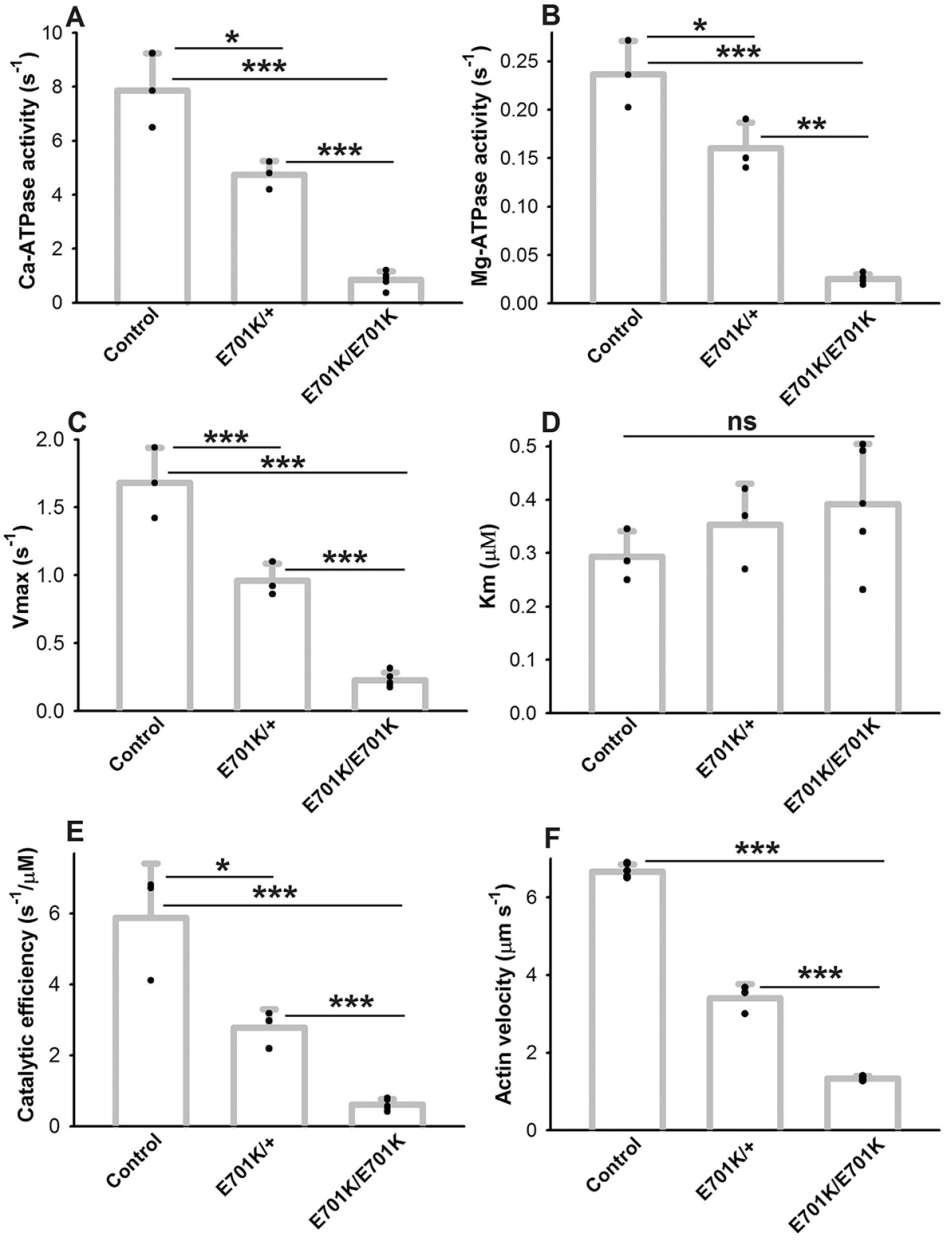
**ATPase activities and *in vitro* motility values for myosin from *E701K*/+ heterozygotes are intermediate between those of homozygotes and wild-type controls.** (A) CaATPase activities. The value for the *E701K*/+ heterozygote is significantly different from both *PwMhc2*/+ control and *E701K*/*E701K* homozygotes. (B) MgATPase activities. The value for the *E701K*/+ heterozygote is significantly different from both control and *E701K*/*E701K* homozygotes. (C) *V_max_* for actin-activated MgATPase activities. The value for the *E701K*/+ heterozygote is significantly different from both control and *E701K*/*E701K* homozygotes. (D) *K_m_* values are actin concentrations at which half-maximal actin-activated MgATPase activities (*V_max_*) are exhibited. No significant differences are exhibited among the samples. (E) Catalytic efficiency (ratio of *V_max_* to *K_m_*). The value for the *E701K*/+ heterozygote is significantly different from both control and *E701K*/*E701K* homozygotes. (F) *In vitro* velocity of actin filaments propelled by myosins of each genotype. The value for the *E701K*/+ heterozygote is significantly different from both control and *E701K*/*E701K* homozygotes. In all *E701K/E701K* homozygote assays, *n*=4. *n*=3 for all other samples, except control *in vitro* motility (*n*=5). *E701K/E701K* homozygote data median values and wild-type motility median values are from Wang et al. (2012). Each ATPase data point is a biological replicate that is the mean of duplicate technical replicates. *In vitro* motility biological replicates represent the mean of over 20 actin filaments per sample. Statistical significance was measured using Student's *t*-test (*P<0.05; **P<0.01; ***P<0.001; ns=not significant). All values are mean±s.d.

We previously reported that the IBM3 mutation induced poor basal and actin-activated ATPase activity, as well as a significant reduction in catalytic efficiency in the fast IFM myosin isoform (Wang et al., 2012). As myosin isolated from *E701K*/*+* IFMs consists of a population of E701K mutant and wild-type molecules, we predicted that biochemical properties would be the average for those of wild-type IFM myosin and pure E701K myosin. Alternatively, it is possible that the instability of the mutant protein in the heterozygote could lead to activity levels nearer to wild type. To examine this, we compared the ATPase parameters of myosin isolated from *E701K*/*+* IFMs and from *PwMhc2*/*+* control IFMs (±s.d.) (Fig. 1) with values defined for myosin from *E701K* homozygotes (Wang et al., 2012). CaATPase activity (Fig. 1A) of pure E701K myosin was roughly 11% of the wild-type control (0.84±0.36 versus 7.86±1.38 s^−1^, *P*<0.001), whereas E701K/+ myosin activity increased to 60% of the control (4.74±0.51 s^−1^, *P*<0.05 and *P*<0.001 compared with both wild type and homozygotes, respectively). Basal MgATPase activity (Fig. 1B) of pure E701K myosin was ∼13% of the control (0.03±0.01 versus 0.23±0.04 s^−1^, *P*<0.001), whereas E701K/+ myosin increased the basal activity to nearly 70% of the control value (0.16±0.03 s^−1^, *P*<0.05 and *P*<0.001 compared with both wild type and homozygotes, respectively). Actin-activated *V_max_* of myosin (Fig. 1C) from the E701K homozygotes was ∼14% of the control (0.23±0.06 versus 1.68±0.26 s^−1^, *P*<0.001), whereas E701K/+ myosin was ∼57% of the control (0.96±0.13 s^−1^, *P*<0.001 compared with both wild type and homozygotes). *K*_*m*_ values (Fig. 1D), the actin levels required for half maximal ATPase stimulation, were not significantly changed in pure E701K myosin compared with control (0.39±0.11 versus 0.29±0.05 μM), with the E701K/+ myosin yielding a value that did not differ significantly from either wild type or homozygotes (0.32±0.08 μM). Catalytic efficiency (*V_max_*/*K_m_* ratio; Fig. 1E) was reduced in pure E701K myosin to ∼11% of control (0.61±0.16 versus 5.88±1.53 s^−1^ µM^−1^, *P*<0.001). In E701K/+ myosin, it improved to nearly 49% of the control value (2.78±0.52 s^−1^ µM^−1^, *P*<0.05 compared with wild type, *P*<0.001 compared with homozygote). Overall, myosin isolated from *E701K*/+ heterozygotes yielded enzymatic activity values that are roughly the calculated mean of the wild-type control and the *E701K* homozygote levels (CaATPase: 4.74 versus 4.35 s^−1^ mean; basal MgATPase: 0.16 versus 0.13 s^−1^ mean; *V_max_*: 0.96 versus 0.96 s^−1^ mean; catalytic efficiency: 2.78 vs 3.17 s^−1^ µM^−1^ mean). This demonstrates that IBM3 myosin and wild-type myosin accumulate at approximately equimolar levels in IFMs from *E701K*/*+* flies.

### *In vitro* motility of actin filaments is reduced for myosin from E701K/+ heterozygotes

We used *in vitro* actin sliding assays to determine the unloaded velocity of fluorescently labeled actin filaments generated by E701K, E701K/+ and PwMhc2 transgenic control myosin (±s.d.) (Fig. 1F). We have previously shown that E701K myosin translocates fluorescently labeled actin filaments at 20% of the velocity of wild-type fast muscle myosin (1.34±0.05 versus 6.67±0.18 µm s^−1^; *P*<0.001) (Wang et al., 2012). Myosin isolated from *E701K*/*+* heterozygote IFMs moves actin filaments at a velocity of 3.41±0.36 µm s^−1^, amounting to 51% of wild-type velocity (*P*<0.001 compared with both wild type and homozygotes). This is ∼15% below the average velocity of the wild-type and E701K homozygote myosins (4.00 µm s^−1^). The disparity in actin filament velocity variance is graphically depicted in Fig. S1.

### Ultrastructural analysis shows generally normal myofibril assembly followed by moderate structural degeneration in *E701K*/+ IFMs

**Fig. 2.**
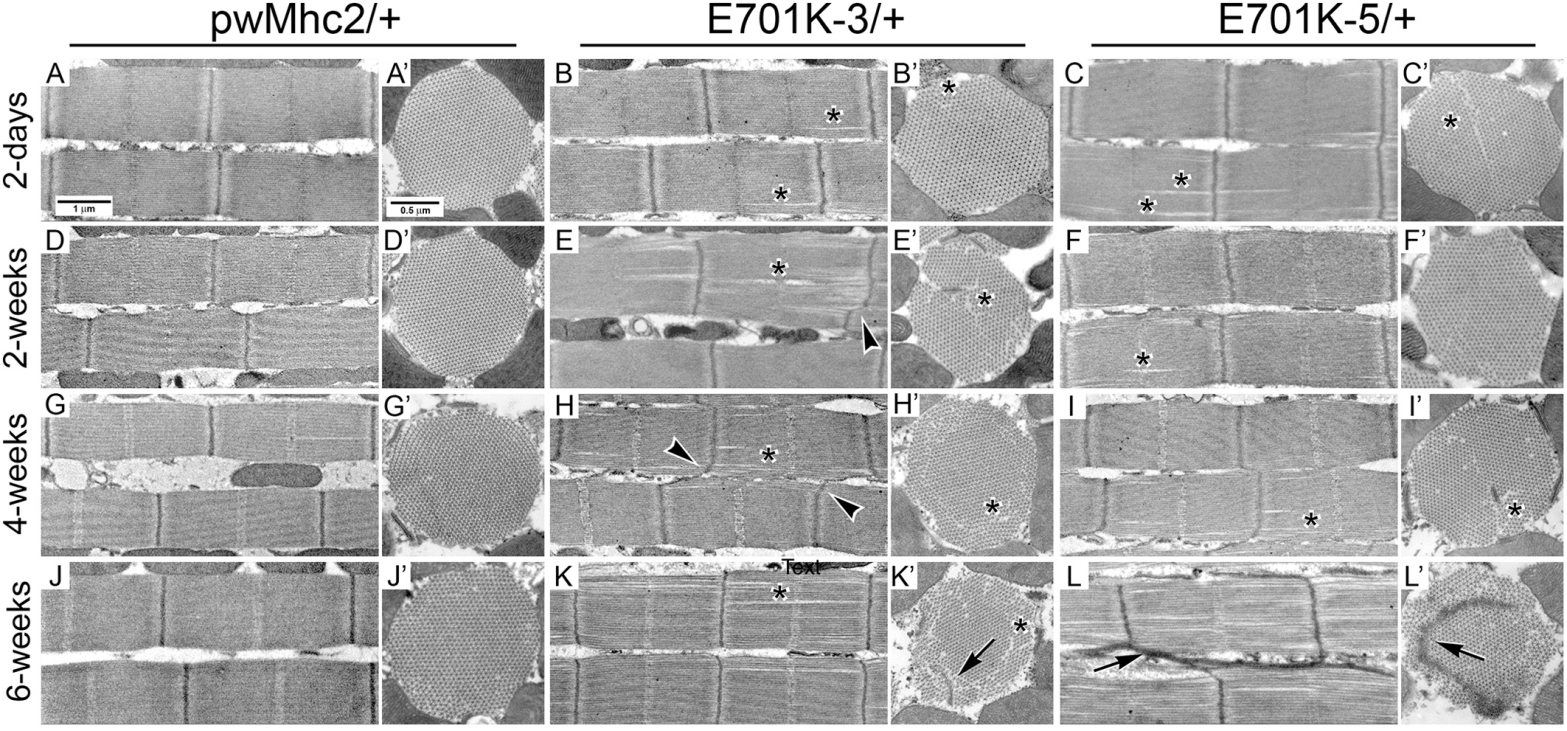
**Ultrastructure of myofibrils from control and *E701K*/+ heterozygote IFMs during aging shows progressive defects in filament arrangement and Z-line organization.** Transmission electron micrographs of longitudinal and transverse sections through IFM myofibrils from adult flies aged 2 days, 2 weeks, 4 weeks or 6 weeks after eclosion. *PwMhc2* wild-type transgenic controls (A,A′,D,D′,G,G′,J,J′) assemble into well-organized sarcomeres (A) and this structure is retained as the flies age (D,G,J). *PwMhc2* myofilaments are packed in a rigid double hexagonal array (A′) that is consistent during aging (D′,G′,J′). Two-day-old myofibrils from *E701K-3*/+ and *E701K-5*/+ IFMs also assemble well-ordered sarcomeres (B,C) with double-hexagonal filament packing (B′,C′), although occasional minor gaps in the microfilament arrays are observed (asterisks). These gaps are exacerbated during aging in longitudinal sections (E,F,H,I,K,L) and are particularly evident in transverse sections (E′,F′,H′,I′,K′,L′). As the heterozygotes age, Z-lines become non-linear (arrowheads), and Z-line streaming, where Z-line material is mislocalized, is observed (K′,L,L′; arrows). Scale bars: 1 µm (longitudinal sections); 0.5 µm (transverse sections).

To determine the effect of heterozygous expression of IBM3 myosin on IFM myofibril assembly and stability, we studied age-matched control and *E701K*/+ mutant IFMs using transmission electron microscopy (Fig. 2). Longitudinal sections of both heterozygotes (*E701K-3* and *E701K-5*) revealed occasional gaps in the myofibrillar lattice at 2 days of age (Fig. 2B,C). These aberrations occurred with increasing frequency as flies aged from 2 days through 6 weeks, as did defects in Z-lines, including non-linearity and abnormal sarcomeric localization (Fig. 2E,F,H,I,K,L). Such defects were not observed in the *PwMhc2*/*+* wild-type control (Fig. 2A,D,G,J). Transverse views of myofibrils from adults at 2 days of age revealed that some *E701K*/+ myofibrils had minor disruptions, with occasional gaps in the myofilament lattice (Fig. 2B′,C′). These defects increased during the aging process (Fig. 2E′,F′,H′,I′,K′,L′), as did the propensity for mislocalization of Z-line material to the filament-containing region of the myofibril (Fig. 2K′,L′). Notably, we saw neither the rapid ultrastructural degradation nor the vacuoles and autophagic vesicles that we had found in IBM3 homozygotes (Wang et al., 2012). Although homozygote IFMs were too structurally damaged to allow flight or permit mechanical analysis, the nearly wild-type structure of heterozygote IFMs at 2 days of age allowed us to assess flight performance and determine fiber mechanical parameters.

### Locomotion assays reveal reduced wing beat frequency and decreased flight ability in *E701K*/*+* heterozygotes

We examined the wing beat frequency of female IBM3 heterozygotes (*E701K-3*/*+* and *E701K-5*/*+*) at 2-3 days of age. Values for both *E701K*/*+* heterozygous lines significantly decreased by ∼25% compared with control fly values at both 15°C and 25°C (Table 1). In contrast to homozygous IBM3 mutant flies, which demonstrate no flight ability (Wang et al., 2012), *E701K*/*+* heterozygotes exhibited weak flight. Flight indices were significantly decreased by 75% and 57% compared with control values at 15°C and 22°C, respectively (Table 1). We also examined the flight ability of *E701K*/*+* flies during aging at 22°C and found progressive disability (Table 1), with statistically significant reductions of 20% at 1 week (*P*<0.01, Student's *t*-test) and a further reduction of 36% at 3 weeks (*P*<0.001). In contrast, the control line showed no decline at 1 week and no statistically significant decrease over the entire period.

**Table 1.**
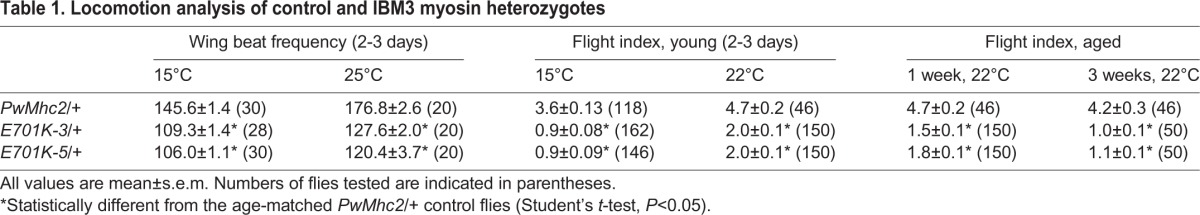
**Locomotion analysis of control and IBM3 myosin heterozygotes**

### Mechanical analysis of *E701K*/+ fibers reveals reduced fiber stiffness, lower power output and altered myosin kinetics

**Fig. 3.**
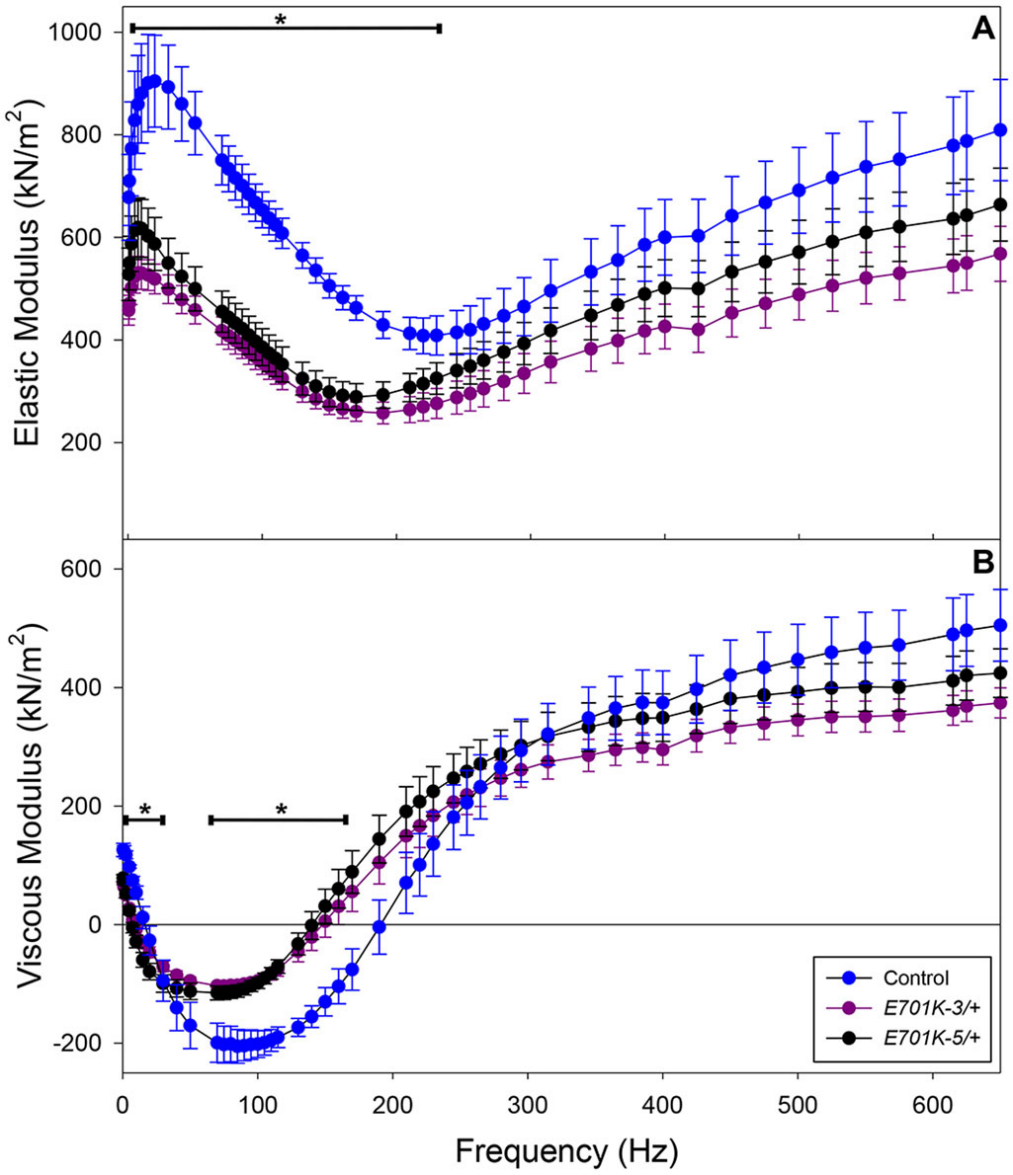
**The effect of muscle length oscillation frequency on elastic modulus and viscous modulus of IBM3 myosin heterozygote IFM fibers differs from control fibers.** (A) Elastic modulus and (B) viscous modulus of control (*PwMhc2*/+) and IBM3 mutant (*E701K-3*/+ and *E701K-5*/+) IFM fibers from 0.5 to 650 Hz. Muscle length change was 0.25% peak to peak. Experiments were performed at 15°C. Mean±s.e.m. *n*=7 for each genotype. Asterisks and horizontal lines indicate the frequencies at which the values show statistically significant differences between the mutant heterozygote and control fibers; one-way ANOVA, *P*<0.05.

To determine the effect of the E701K mutation on muscle viscoelastic properties, we used small-amplitude sinusoidal analysis. Using these results, we calculated elastic modulus (in-phase stiffness) and viscous modulus (out-of-phase stiffness) for the mutant heterozygote and control lines. The elastic modulus was significantly reduced in the mutant lines over the frequency range of 5-220 Hz, with the largest reduction occurring around 140 Hz (Fig. 3A). Comparing dip frequency values (lowest value from all frequencies tested) between mutant heterozygote and control lines, the mutant's elastic modulus, E_e_, was about 40% lower than the control value (Table 2). These in-phase stiffness results suggest that fewer heads are bound to actin (lower duty ratio) or that there is less force generation per head. The viscous modulus was significantly less negative at the lowest frequencies (0.5-1.5 Hz) and also at 70 to 170 Hz (Fig. 3B). At the lowest viscous modulus values, E_v_, the viscous modulus values of the mutant lines were about 50% less than the control (Table 2). As viscous modulus is inversely proportional to work performed by the muscle, less work was generated by the mutant heterozygote fibers at these frequencies, which includes the wing beat frequency range of *Drosophila* at 15°C (Table 1). The fiber mechanical assays were also performed at this temperature.

**Table 2.**
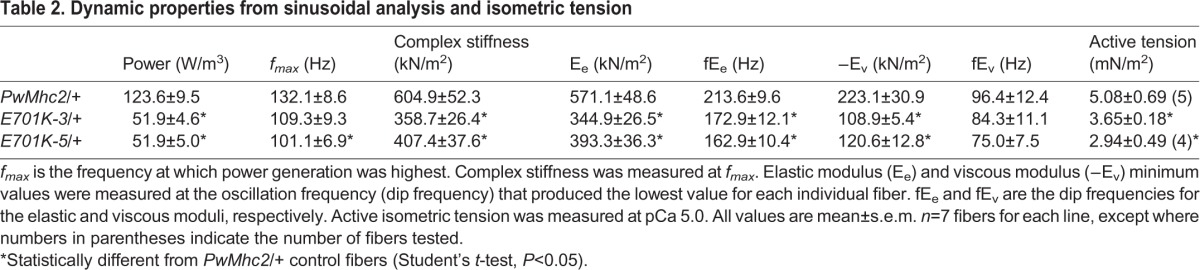
**Dynamic properties from sinusoidal analysis and isometric tension**

**Fig. 4.**
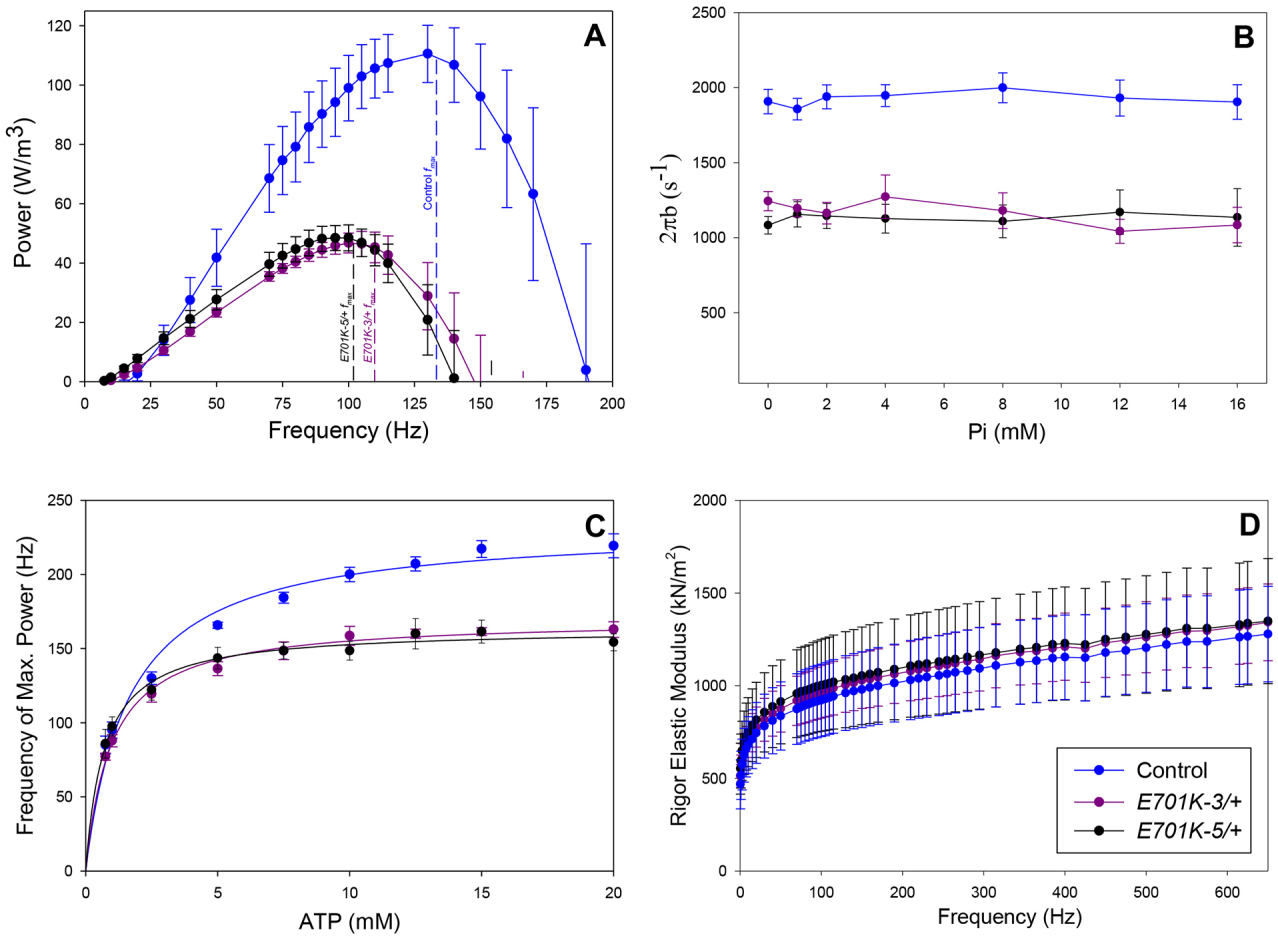
***E701K*/+ heterozygote IFM fibers and control fibers display differences in power output parameters, but not in rigor stiffness.** (A) The power generated by maximally activated control (*PwMhc2*/+) and IBM3 mutant heterozygote (*E701K-3*/+ and *E701K-5*/+) IFM fibers when oscillated at 0.25% peak-to-peak strain over a frequency range of 0.5-200 Hz at 15°C. Vertical dashed lines indicate the frequency at which maximum power was generated (*f_max_*). Data are mean±s.e.m., *n*=7 for each genotype. (B) The response of 2πb to phosphate concentration. Data are mean±s.e.m., *n*=7. (C) Effect of MgATP concentration on the frequency at which maximum power (*f_max_*) is produced in control (*PwMhc2*/+) and IBM3 mutant (*E701K-3*/+ and *E701K-5*/+) IFM fibers. Data are mean± s.e.m., *n*=7. (D) Rigor stiffness determined by measuring elastic modulus at pCa 4.5 and ATP=0 mM from 0.5 Hz to 650 Hz. No significant differences in rigor elastic modulus were observed between the mutant and control fibers; one-way ANOVA, *P*<0.05. Data are mean±s.e.m., *n*=6 for each mutant heterozygote and *n*=5 for control.

Sinusoidal analysis also showed that the mutation decreased muscle power generation and shifted optimal power production to lower frequencies (Fig. 4A). Power was reduced by 59% and *f_max_* was reduced by about 20% due to the mutation (Table 2). Power generated by the mutant heterozygote was almost zero at the wing beat frequency at which control flies beat their wings (∼145 Hz, at 15°C). The decrease in *f_max_* suggests that the mutation slowed overall muscle and myosin kinetics. This slowing of kinetics was supported by the elastic and viscous modulus results. Both mutant modulus graphs show a leftward shift in their dip frequencies, fE_e_ and fE_v_ (lowest values), compared with the control (Fig. 3 and Table 2).

To gain insight into how the cross-bridge cycle might be altered, we determined the influence of the E701K mutation on apparent muscle mechanical rate constants and amplitudes. This was carried out by fitting the complex modulus equation that we previously refined for *Drosophila* IFMs to Nyquist plots of the complex modulus (Swank et al., 2006). The mutation significantly changed these rate constants and their associated amplitudes (Table 3). Based on interpretations from previous studies using this analysis (Kawai and Brandt, 1980; Miller et al., 2010; Palmer et al., 2007), we interpreted the changes as follows (Fig. S2): (1) the decrease in the A process suggests a decrease in stiffness of the passive viscoelastic components of the muscle, potentially including myosin; (2) the decrease in rate constant 2πb suggests that cross-bridge transition rates involving myosin attachment to actin work production are slowed, whereas the increase in 2πc suggests rate constants associated with work absorption and myosin detachment from actin have increased; (3) the amplitude of work-producing steps (B process) and absorbing steps (C process) of the cycle are both reduced, suggesting a decrease in the number of myosins producing force at any given time and/or cross-bridge stiffness during strongly bound steps of the cycle.

**Table 3.**
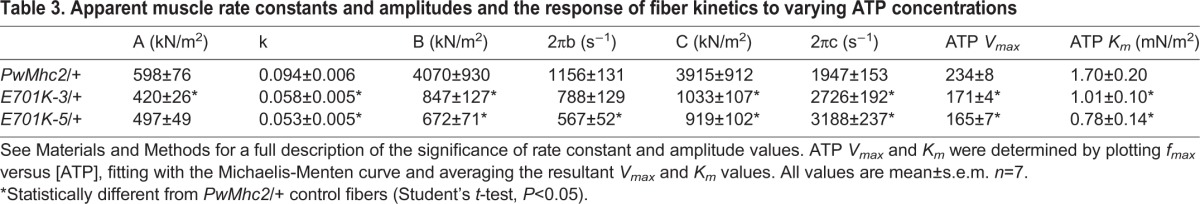
**Apparent muscle rate constants and amplitudes and the response of fiber kinetics to varying ATP concentrations**

We gained additional insight into cross-bridge kinetics by varying ATP and Pi concentrations in conjunction with sinusoidal analysis. Varying Pi concentration did not alter 2πb values of the control or mutants fibers (Fig. 4B). According to our previous modeling (Swank et al., 2006), this suggests the rate-limiting cross-bridge step for work production was not changed by the mutation. Plotting [ATP] versus *f_max_* and fitting the graph with a Michaelis-Menton curve showed that, in addition to decreasing *f_max_*, the mutation reduced ATP *K_m_* values by 50% (Fig. 4C and Table 3). The reduced *K_m_* values suggest an increase in ATP affinity.

The changes in 2πb, 2πc and ATP *K_m_*, suggest a decrease in myosin duty ratio. A decrease in duty ratio, the time myosin spends strongly bound to actin, would be predicted to cause a decrease in isometric tension generation. We observed that active isometric tension was decreased by about 35% (Table 2). It is alternatively possible that the decrease in tension resulting from the mutation could arise from changed cross-bridge stiffness. Measuring the elastic modulus of the fibers in the absence of ATP (rigor conditions where all myosin heads are bound to actin) should indicate a change in cross-bridge stiffness. We did not observe a change in rigor elastic modulus in the mutant heterozygote fibers relative to control (Fig. 4D).

### Effects of increased or decreased *E701K*:wild-type gene ratio

**Fig. 5.**
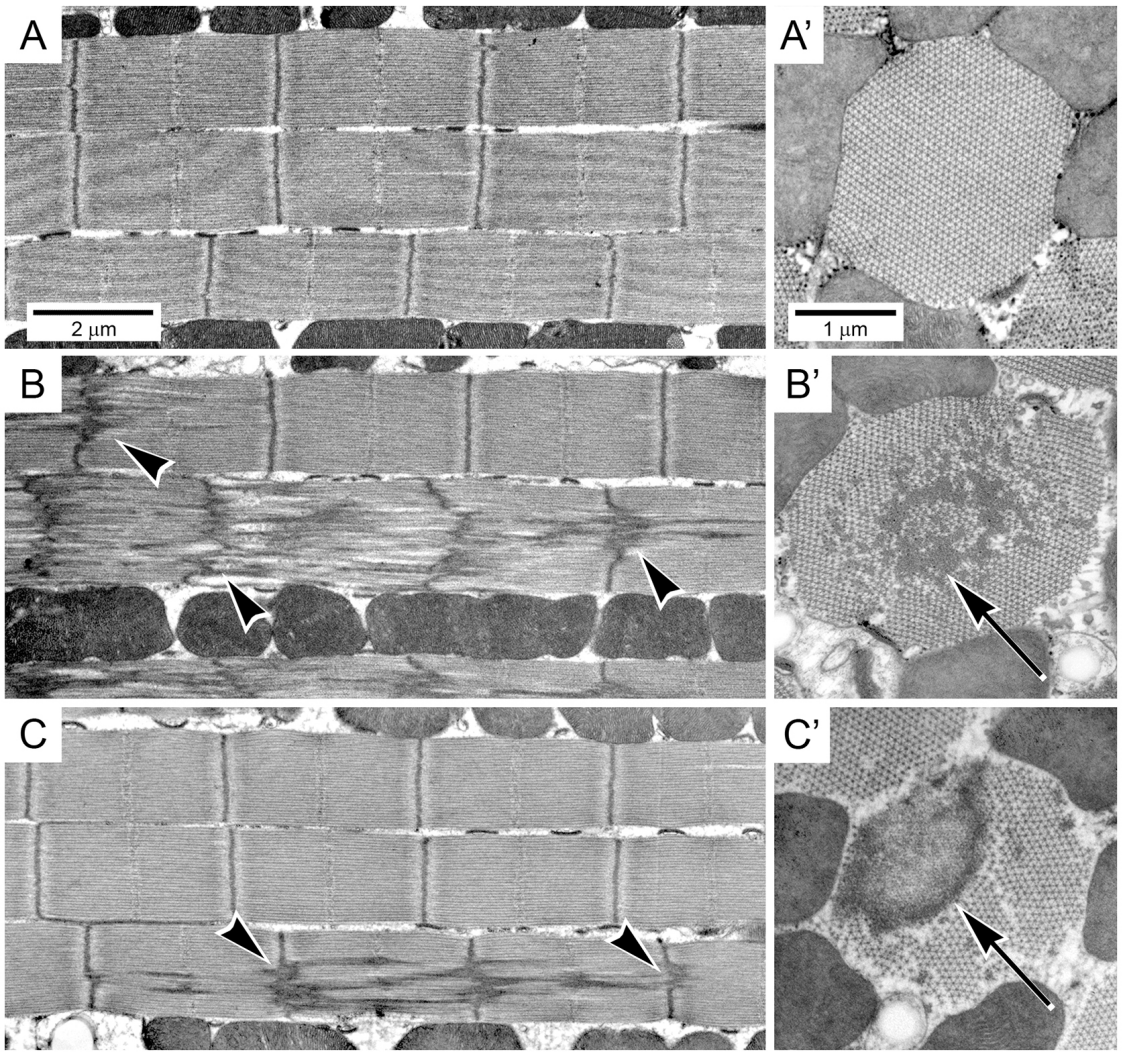
**Ultrastructure of myofibrils expressing two IBM3 *E701K Mhc* genes and one wild-type *Mhc* gene show enhanced myofibrillar degeneration and Z-line streaming.** Transmission electron micrographs of longitudinal and transverse sections through IFM myofibrils from 4-week-old adult flies. *PwMhc2*/*PwMhc2*/+ transgenic control myofibrils (top row) contain well-organized sarcomeres (A), with a double-hexagonal lattice of thick and thin filaments (A′). Myofibrils from (B) *E701K-3*/*E701K-3*/+ and (C) *E701K-5*/*E701K-5*/+ IFMs show severely disordered sarcomeres with non-linear Z-lines (arrowheads) and Z-line streaming. Although some normally structured sarcomeres are present, the overall phenotype is dramatically worsened compared with mutant heterozygotes (Fig. 2). In transverse sections (B′,C′) myofibrillar disorder and mislocalization of Z-line material is evident (arrows). Scale bars: 2 µm (longitudinal sections); 1 µm (transverse sections).

We genetically increased the ratio of mutant (*E701K*) to wild-type (+) myosin alleles to 2:1, in an attempt to discern the ‘tipping point’ for production of high levels of myofibrillar disarray and vesicle formation found in both the IBM3 human heterozygotes and in the *Drosophila* homozygotes. Increased relative gene copy number of E701K myosin resulted in poor flight ability compared with a control line with three copies of wild-type myosin. The average flight index of *E701K/E701K*/+ was reduced by ∼87% compared with *PwMhc2/PwMhc2*/+ at all ages tested (Table S1). This compares with an average of a 65% reduction in flight ability in *E701K* heterozygotes relative to control heterozygotes (Table 1). The ultrastructure of 4-week-old flight muscle revealed a dramatic increase in the frequency of Z-line disruption and focal discontinuities in the myofilamentous lattice (Fig. 5) compared with heterozygous *E701K*/+ muscle at the same age (Fig. 2). However, in contrast to flies expressing only E701K myosin (Wang et al., 2012), the aged IFMs did not display obvious vacuole or autophagic vesicle formation. When the ratio of mutant (*E701K*) to wild-type (+) myosin alleles was altered to 1:2, flight ability was essentially unchanged compared with the three-copy control (Table S1), further indicating that the dose of the mutant allele is directly correlated to the severity of the mutant phenotype. This suggests that increased expression of endogenous or transgenic wild-type myosin genes could prove therapeutic in IBM3 patients.

## DISCUSSION

We modeled the dominant effects of the human IBM3 myosin mutation in *Drosophila* muscle and obtained insights into the basis of both the mechanical and structural defects caused by this mutation. Our data indicate that equimolar levels of mutant myosin accumulate in young heterozygotes, in agreement with the transcriptional level results in human patients (Tajsharghi et al., 2002). We observed a 51% decrease for *in vitro* motility of actin filaments driven by myosin isolated from *Drosophila* heterozygotes, which mimics the 56% reduction observed in myosin isolated from biopsies of humans expressing the mutation (Li et al., 2006). Despite essentially normal myofibrillar structure in young *Drosophila* IFMs, fiber power output and muscle function are dramatically reduced. During aging, we observed myofibrillar degeneration, including Z-disc streaming, accompanied by a further reduction in muscle function. The progressive nature of the *Drosophila* IBM3 heterozygote phenotype mimics the decreased muscle function and increased myofibril disarray observed in aging human heterozygotes (Tajsharghi et al., 2002).

Human IBM3 disease presentation is complicated compared with *Drosophila*, in that multiple fiber types are present and co-expression of myosin isoforms occurs in human mixed fiber muscles (Schiaffino and Reggiani, 1994). In individuals with IBM3, increased fiber size variability as well as increases in mass and function of non-type IIa fibers have been documented (Li et al., 2006; Tajsharghi et al., 2002), likely as being compensatory for the abnormalities found in type IIa fibers. Cross-sectional area measurements of dorsolongitudinal IFM fibers in IBM3 heterozygotes did not show a statistically significant difference compared with control organisms at 2-3 days of age (Table S2), indicating a lack of hypertrophic compensation. As *Drosophila* does not have the ability to undergo fiber growth through use of satellite stem cells (Demontis et al., 2013) or through activation of other myosin heavy chain genes (Bernstein et al., 1983; Rozek and Davidson, 1983), this result is not surprising. Although the *Drosophila* system does not mimic all properties of human skeletal muscle, it has the ability to directly define the biochemical and mechanical origins of the disease phenotype. *Drosophila* has the added advantage of genetic homogeneity that is lacking in human patients who often display different disease severities, likely arising from disparate modifying alleles (Li et al., 2006).

Previous work that modeled the IBM3 mutation in *Dictyostelium* non-muscle myosin II (Zeng et al., 2004) suggested that product release rate, particularly that of ADP, is reduced as a result of the SH1-SH2 helix mutation. This would result in myosin spending increased time bound to actin, yielding a higher duty ratio (the fraction of the chemomechanical cycle in which actin is bound to myosin). This could explain the slower actin motility we observed with both homozygous and heterozygous mutant myosin, and would be in keeping with the reduced overall muscle kinetics that yielded a reduced *f_max_*.

A number of our findings, however, do not support a model for IBM3 muscle myosin spending increased time in strongly actin-bound states. In contrast to the increases in active stiffness and active force generation expected for augmented actin binding, we found that both elastic and viscous modulus decreased (Fig. 3), and that isometric force generation was either unchanged or reduced (Table 2). Additionally, our sinusoidal analysis uncovered an increase in the apparent rate constant 2πc (Table 3), a parameter that is inversely proportional to time strongly bound to actin (Kawai and Brandt, 1980; Palmer et al., 2007). This suggests that the mutant myosin spends less time in strongly bound states, such as the ADP state. Furthermore, varying ATP concentration while performing sinusoidal mechanical analysis revealed a slightly higher affinity of the heterozygous myosin for ATP, again suggesting faster transitions through the final steps prior to myosin dissociation from actin. Finally, we did not observe a dramatic reduction of *in vitro* actin filament velocity in heterozygote myosin below the average of levels for the control and mutant homozygote, despite the fact that our ATPase activity assays indicate the presence of equimolar levels of IBM3 and wild-type myosin (Fig. 1). As equimolar mixtures of skeletal muscle myosins (which are regulated by ADP off-rate) translocate actin at the rate of the slower isoform (Cuda et al., 1997; Sata et al., 1993), our observations suggest that the mechanical results do not arise from an increase in the time myosin spends strongly bound to actin.

Our data instead support a mechanism in which the power stroke itself or the rates of steps associated with the power stroke have been altered in the IBM3 heterozygote, so that fewer or shorter power strokes occur. The IBM3 mutation severely decreased rate constant 2πb (Table 3), which is influenced primarily by work-generating steps associated with actin attachment and the power stroke. In concordance with this, we observed no change in the response of 2πb to Pi concentration for the heterozygote (Fig. 2B). If ADP release rate had been substantially decreased, as suggested above, then it would have become rate limiting, and 2πb should have increased with Pi concentration (Kawai and Brandt, 1980). In addition, the decreased rate of actin translocation in the *in vitro* motility assay would be expected to yield a more linear relationship between motility and isoform level if fewer or shorter power strokes result from the mutation, compared with when the slower IBM3 myosin remains bound for a longer period due to a reduced ADP release rate. Finally, and in agreement with these conclusions, the observed lower B, C and stiffness values for the IBM3 heterozygotes (Tables 2 and 3) suggest that less myosin is bound at any given time. The observed lower stiffness and force in the heterozygote do not arise from reduced cross-bridge stiffness, as rigor stiffness was not changed in our assays (Fig. 4D).

The slower kinetics and less-stiff IFM fibers explain the decrease in flight ability observed in IBM3 heterozygotes (Table 1). Because a decreased elastic modulus correlates with decreased wing beat frequency (WBF) (Ramanath et al., 2011), the lower active stiffness caused a lower WBF in the mutants (Table 1), reducing aerodynamic power for flight. Attempts by IBM3 heterozygotes at beating their wings at a higher frequency would provide inadequate power for flight, as muscle power output for the mutant heterozygote is negligible at the wild-type WBF (Fig. 4A).

The modest ultrastructural degradation observed in the IBM3 heterozygote (Fig. 2) is in consonance with a mechanism for IBM3 that involves a reduced power stroke size and/or number. The resulting reduced duty ratio should not be as detrimental to ultrastructure as an increase in the time spent in strongly bound actin states, as is the case when an embryonic isoform of myosin is expressed in IFMs (Wells et al., 1996) or when unregulated thin filaments are present (Beall and Fyrberg, 1991). A single copy of the wild-type myosin gene is even sufficient to permit relatively normal ultrastructure to be maintained in the presence of two *IBM3* myosin alleles (Fig. 5). Furthermore, well-aligned thick filaments were seen in an IBM3 *C. elegans* model that co-expresses a wild-type isoform of myosin heavy chain (Tajsharghi et al., 2005). The ‘protective’ effects of wild-type myosin are seen in human patients, where focal disruptions in ultrastructure occur, rather than overall sarcomere degradation (Martinsson et al., 2000; Oldfors, 2007). By contrast, the more severe ultrastructural abnormalities observed in IBM3 homozygotes (Wang et al., 2012), which are not capable of producing mechanical power output (data not shown), are likely to arise from serious defects in assembly due to the lack of wild-type myosin molecules.

As in the human condition, where functional defects precede structural abnormalities (Li et al., 2006), *Drosophila* IBM3 heterozygotes show exacerbation of muscle structural and functional defects during aging (Fig. 2, Table 1). Most notably, focal defects in sarcomere structure with streaming Z-line material were observed in both the model and the disease state (Martinsson et al., 2000; Oldfors, 2007). It is possible that this arises when functional defects cause abnormal stress on myofibrillar components, resulting in structural degradation. However, we did not detect an increase in the stress response in the heterozygote. Levels of the protein aggregate marker Ref(2)P (p62 in mammals) and the ubiquitin protein degradation signal are not increased in heterozygotes compared with controls (J.A.S., G.C.M., A.M., E. P. Ratliff, D. B. Foster and S.I.B., unpublished data), although levels of these proteins increase in IBM3 homozygotes (Suggs et al., 2015). Previous studies have shown that Ref(2)P levels increase during both stress and aging in *Drosophila*, and that ubiquitin levels increase during muscle aging (Demontis and Perrimon, 2010; Dialynas et al., 2015; Landis et al., 2012). As aging leads to structural deterioration in *Drosophila* IFMs (Miller et al., 2008), it is possible that other aspects of the aging response (reviewed by Demontis et al., 2013) are triggered by the E701K myosin mutation, leading to the observed phenotypic abnormalities. Another possibility is that the mutant myosin is more prone to proteolysis, possibly as a result of its tendency to aggregate (Wang et al., 2012). Tajsharghi et al. speculated that proteolytic degradation products could contribute to toxic effects on muscle structure, as they observed a disproportionally high level of myosin IIA mRNA compared with protein levels in patients, suggesting increased protein turnover (Tajsharghi et al., 2002). In contrast to the myofibrillar defects observed in the *Drosophila* heterozygote that mirror the human condition, we did not discern the rimmed vacuoles observed in patients. It is notable, however, that such structures are not detected in all human type IIa muscles, being most prevalent in older patients who are more seriously disabled (Martinsson et al., 2000; Tajsharghi et al., 2002).

The location of the IBM3 mutation within the SH1-SH2 helix places it in proximity to the nucleotide binding pocket, to the central transducer region that communicates with the actin-binding site and to the relay domain that is important for inducing lever arm movement through interaction with the converter domain. Alterations of the SH1-SH2 helix, such as crosslinking of the active sulfhydryl residues (Thompson et al., 2008) or various mutations (Hu et al., 2002; Kad et al., 2007; Preller et al., 2011; Zeng et al., 2004) affect ATP hydrolysis and/or transduction of its chemical energy into the power stroke. In accordance with these observations, we detected decreased ATPase rates in mutant myosin (Fig. 1). This could arise from a decrease in rate-limiting Pi release. This would correlate with the observed reduction in 2πb and lead to a decrease in length or probability of occurrence of the power stroke. Our previous modeling results (Wang et al., 2012) predicted that the melted state of the SH1-SH2 helix that occurs after the power stroke and uncouples the lever arm from the motor (Himmel et al., 2002) is stabilized by the IBM3 mutation. This abnormal interaction could indeed decrease the probability of transitioning through the power stroke or reduce its size, as predicted from our mechanical data. Based upon our findings, pharmacological agents that enhance the kinetics of myosin binding to actin and steps of the cycle associated with the power stroke, might be expected to improve the functioning of type IIa-containing fibers in young IBM3 patients, and perhaps reduce the structural and functional degradation that occurs during aging.

## MATERIALS AND METHODS

### Transgenic lines and genetic crosses

The *Drosophila melanogaster E701K* mutation in the *Mhc* gene was created by site-directed mutagenesis of an *E. coli* plasmid and introduced into the fly genome by *P* element-mediated germline transformation along with a *w^+^* eye color marker (Wang et al., 2012). The transgene was crossed into the *Mhc^10^* genetic background, which contains a null allele that results in flies lacking myosin expression in the indirect flight and jump muscles (Collier et al., 1990). Two mutant fly lines homozygous for *Mhc^10^* and the E701K transgene on the third chromosome (*w*; *Mhc^10^*; *E701K-3* and *w*; *Mhc^10^*; *E701K-5*) were shown to express wild-type levels of E701K protein in the IFMs (Wang et al., 2012). A line carrying a transgene encoding full-length wild-type myosin heavy chain on its first chromosome, *PwMhc2*; *Mhc^10^*, is used as a control (Swank et al., 2000).

For studies of heterozygotes, the lines outlined above were each crossed with a ‘wild-type’ *yw* fly line to create progeny with one wild-type endogenous *Mhc^+^* allele, one *Mhc^10^* allele and one copy of the respective transgene, i.e., *w*, *PwMhc2*/*yw*; *Mhc^10^*/+ for the control line; *w*/*yw; Mhc^10^*/*+*; *E701K-3*/*+* and *w*/*yw; Mhc^10^*/*+*; *E701K-5*/*+* for the two IBM3 heterozygous lines. Female progeny were aged and used for subsequent experiments.

In addition to analyzing heterozygotes with 1:1 *E701K*:*Mhc*^+^ genotypic ratio, we analyzed flies with 1:2 or 2:1 *E701K*:*Mhc*^+^ genotypic ratios. The former were produced by crossing male *yw*; *Mhc^+^*/*Mhc^+^*; *E701K*/*E701K* flies to female *yw* flies to create *yw*; *Mhc^+^*/*Mhc^+^*; *E701K/+* progeny. Similarly, control male *yw PwMhc2*; *Mhc^+^*/*Mhc^+^* flies were crossed with female *yw* to create *yw PwMhc2*/*yw*; *Mhc^+^*/*Mhc^+^* control progeny. Flies with a 2:1 *E701K*:*Mhc*^+^ genotypic ratio were produced by crossing male *yw*; *Mhc^+^*/*Mhc^+^*; *E701K*/*E701K* flies to female *w*/*w*; *Mhc^10^*/*Mhc^10^*; *E701K*/*E701K* flies, yielding *w*/*yw; Mhc^10^*/*Mhc^+^*; *E701K*/*E701K*. These flies were compared with control flies with two transgenic wild-type myosin genes and one endogenous gene, *PwMhc2*/*PwMhc2*; *Mhc^10^*/ *Mhc^+^*.

### Protein expression

Relative levels of myosin expression were determined by measuring myosin-to-actin ratios in upper thorax homogenates subjected to one-dimensional SDS polyacrylamide gel electrophoresis (O'Donnell et al., 1989). Six dissected upper thoraces from 2-day-old adult female flies for each line were homogenized in 360 μl of SDS gel 1× Laemmli Sample Buffer (Bio-Rad, Hercules, CA) containing 5% 2-mercaptoethanol. Samples were boiled for 5 min prior to loading at 2-5 μl on a 15-well 10% Mini-PROTEAN TGX polyacrylamide gel (Bio-Rad). Gels stained with Coomassie Brilliant Blue R-250 were digitally scanned using an Epson Expression 636 flatbed scanner. Myosin heavy chain and actin levels were determined by UN-SCAN-IT gel software (Silk Scientific) for three separate lanes per sample. The median myosin-to-actin ratio in mutant thoraces was compared with the median ratio found in thoraces from aged-matched flies expressing the *PwMhc2* control transgene in the corresponding genetic background.

### Protein purification

Myosin was purified from the dorso-longitudinal set of IFMs of ∼120 young female flies as previously described (Kronert et al., 2008, 2014; Swank et al., 2001). Briefly, fibers were collected in York modified glycerol [20 mM potassium phosphate (pH 7.0), 2 mM MgCl_2_, 1 mM EGTA, 20 mM DTT and a protease inhibitor mixture], pelleted and then demembranated for 30 min on ice in York modified glycerol containing 2% (v/v) Triton-X 100. After centrifugation and washing, the solution was replaced with a high-salt extraction buffer [1 M KCl, 50 mM potassium phosphate (pH 6.8), 5 mM MgCl_2_, 0.5 mM EGTA, 10 mM sodium pyrophosphate, 20 mM DTT, plus a protease inhibitor mixture]. The soluble material was subjected to a low-salt precipitation to pellet myosin via centrifugation, a high-salt precipitation to remove residual contaminants and another low-salt precipitation to isolate purified myosin. Protein was suspended in myosin storage buffer [0.5 M KCl, 20 mM MOPS (pH 7.0), 2 mM MgCl_2_ and 20 mM DTT]. Myosin concentration was determined by measuring the absorption at 280 nm (Margossian and Lowey, 1982). A typical yield was ∼100 µg of myosin. Samples were immediately used for ATPase and *in vitro* motility assays.

G-actin was isolated from acetone powder of chicken breast muscle (Pardee and Spudich, 1982) by cycles of polymerization and depolymerization as previously described (Kronert et al., 2014). Following dialysis, actin concentration was determined by absorption at 280 nm. F-actin was prepared by adding one volume of 10× polymerization buffer [50 mM Tris-Cl (pH 8), 0.5 M KCl, 20 mM MgCl_2_, 10 mM ATP] to nine volumes of G-actin. Actin, stored on ice at 4°C, was used within 1 month of preparation.

### ATPase activity

ATPase activities were determined using 2 µg of freshly prepared myosin and 1 mM [γ-^32^P]ATP. Basal ATPase activities were assessed for 15 min in MgATPase buffer (10 mM imidazole, pH 6.0, 20 mM KCl, 2 mM MgCl_2_, 0.1 mM CaCl_2_) or CaATPase buffer (10 mM imidazole, pH 6.0, 0.1 M KCl, 10 mM CaCl_2_) at 25°C. Following sample extraction with 1.8 M HClO_4_, incorporation of radiolabel was determined by scintillation counting. Actin-activated MgATPase activity was assessed by the addition of chicken skeletal muscle actin (0.1–2 μM) to samples prior to incubation. Following subtraction of basal MgATPase activity from each data point, the ATPase activity versus actin concentration was graphed. The Michaelis-Menten equation was used in conjunction with SigmaPlot (Systat Software, San Jose, CA, US) to define *V_max_*, *K_m_* and catalytic efficiency (*V_max_*/*K_m_*) values. Detailed procedures for ATPase activity determination have been previously described (Kronert et al., 2008, 2014; Swank et al., 2001).

### *In vitro* actin sliding velocity

*In vitro* actin sliding velocity assays were implemented as previously described (Kronert et al., 2008, 2014; Swank et al., 2001). Briefly, nitrocellulose-coated coverslips were treated with myosin at 0.5 μg/μl and myosin heads that irreversibly bound to actin were blocked with unlabeled actin filaments. F-actin labeled with fluorescent phalloidin was added to the coverslip and filament movement was digitally captured under fluorescent optics in the presence of ATP. Computational analysis of actin filament movement permitted determination of actin sliding velocity.

### Ultrastructural analysis

Preparation of samples for transmission electron microscopy was carried out as previously described (Suggs et al., 2007; Wang et al., 2012). Samples were viewed on a Tecnai 12 microscope at 120 kV and images were captured with a XR-41S 2k digital camera and software from Advanced Microscopy Techniques.

For muscle fiber measurements, transverse sections at ∼200 nm were collected from 2- to 3-day-old fly thoraces prepared for ultrastructural analysis and embedded in resin as indicated above. Sections were transferred onto a standard glass microscope slide and briefly heated to insure adherence. This was followed immediately by staining in 1% Methylene Blue and 1% borax. Samples were viewed and digitally photographed at 40× under a light microscope. A stage micrometer with 0.01 mm divisions was similarly imaged for scale calibration. Cross-sectional areas of each member of collateral pairs of DLM fibers 45d, 45e and 45f (numbering according to Miller, 1950) from three flies per genotype were measured using ImageJ Version 1.51j (https://imagej.nih.gov/ij/download.html). Mean values were assessed for statistically significant differences using a two-way ANOVA with Dunnett's multiple comparisons.

### Flight assays

Flight assays were conducted on female flies at 15°C, 22°C and 25°C. The flight phenotype was determined by observing whether each fly tested flew upward (U), horizontal (H), down (D) or displayed no flight (N) when released in a flight chamber (Drummond et al., 1991), and quantified using a flight index equal to 6 U/T+4H/T+2D/T+0N/T, where T is the total number of flies tested (Tohtong et al., 1995). Wing beat frequency (WBF) was measured on 2- to 3-day-old female flies using an optical tachometer (Hyatt and Maughan, 1994).

### Fiber mechanics

A pair of IFM bundles containing six fibers each were dissected out of 2- to 3-day**-**old female fly thoraces and demembranated for 1 h at 4°C in skinning solution [5 mM ATP, 1 mM free Mg^2+^, 0.25 mM phosphate, 5 mM EGTA, 20 mM N, N-Bis (2-hydroxyethyl)-2-aminoethanesulfonic acid (BES) at pH 7.0, 1 mM DTT, 50% glycerol and 0.5% Triton X-100; pCa 8.0 and ionic strength 175 mM, adjusted with Na methane sulfonate]. Tungsten wire probes were used to separate and split the individual fibers. An aluminum clip was wrapped around each end of the fiber preparation. The preparations were attached to a mechanical measurement apparatus and bathed in relaxing solution (5 mM ATP, 8 mM phosphate, 15 mM creatine phosphate, 300 U/ml creatine phosphokinase, 1 mM free Mg^2+^, 5 mM EGTA, 20 mM BES at pH 7.0, 1 mM DTT; pCa 8.0; ionic strength 200 mM, adjusted with Na methane sulfonate). Fibers were stretched by 5% from resting length then subjected to sinusoidal analysis (see below) in relaxing solution (pCa 8.0) before a partial exchange with activating solution (same as relaxing solution but pCa adjusted to 4.0) in the chamber to bring pCa to 5.0. The active fiber was further stretched (in 2% increments of fiber length between clips) until maximum power generation was obtained. At this optimized length, different relaxing, activating or rigor solutions were exchanged into the chamber and further sinusoidal analysis, tension measurements or [ATP] and [Pi] variations were performed. Sinusoidal analysis was run at the beginning and end of each experiment to ensure the performance of a fiber did not decrease by more than 10%. Detailed methods have been previously presented (Swank, 2012).

### Sinusoidal analysis and muscle mechanical rate constants

To measure muscle stiffness and mechanical rate constants of the fibers, sinusoidal analysis was performed as described previously (Swank, 2012; Swank et al., 2006). Briefly, a 0.125% muscle length peak-to-peak amplitude sine wave was applied to the fiber at 50 frequencies from 0.5 to 650 Hz. At each frequency, the amplitude ratio and phase difference for force and length were calculated. The ratio was divided by the fiber cross-sectional area to obtain the complex, elastic and viscous moduli.

The complex modulus from each fiber was fitted to a 3-term equation. This equation is based on the equation originally developed by Kawai and Brandt (Kawai and Brandt, 1980) for sinusoidal analysis and has been slightly modified to be more suitable for IFMs (Swank et al., 2006): Y(f)=A (2π if/α)^k^–B if/(b+if)+C if/(c+if), where f is the applied frequency of oscillation (0.5-650 Hz), i is the square root of −1, α is defined as 1 Hz and k is a unit-less exponent (Swank et al., 2006). The first term (A) reflects the viscoelastic properties of passive structures within the fiber, while the second and third terms (B and C) reflect an outcome of transitions between cross-bridge states (changes in complex moduli due to the strain sensitivity of cross-bridge rate constants) that are exponential in the time domain. Process B is primarily influenced by the work-producing steps of the cross-bridge cycle while process C is influenced by the work-absorbing steps prior to and including myosin detachment from actin (Fig. S2). Processes B and C appear as hemispheres in the Nyquist plot with characteristic frequencies b and c (Swank et al., 2006). In the time domain, these frequencies correspond to rate constants 2πb and 2πc. For a more-detailed explanation, including information on the relationship between these rate constants and those derived from step analysis, see Kawai and Brandt (Kawai and Brandt, 1980).

### Pi and ATP concentration experiments

To obtain more information about the effect of the mutation on the cross-bridge cycle rate constants, ATP and Pi concentrations were varied in the bathing solutions. For the Pi experiments, the following activating solution components were adjusted to 13 mM MgATP, 37 mM creatine phosphate, 450 U/ml creatine phosphokinase and an ionic strength of 260 mM. For the ATP experiments, the following activating solution components were adjusted to 0 mM Pi, 37 mM creatine phosphate and 450 U/ml creatine phosphokinase, with an ionic strength of 260 mM. The ATP response was fit with an exponential rise to maximum curve.
